# Proposed task shifting integrated with telemedicine to address uncorrected refractive error in Kenya: Delphi study

**DOI:** 10.1186/s12913-024-10618-8

**Published:** 2024-01-22

**Authors:** Shadrack Muma, Kovin Shunmugam Naidoo, Rekha Hansraj

**Affiliations:** 1 College of Health Sciences, Department of Optometry, University of KwaZulu-Natal, Durban, South Africa; 2 OneSight EssilorLuxottica Foundation, Paris, France

**Keywords:** Task shifting, Human resource, Refractive error, Vision corridors, Telemedicine

## Abstract

**Background:**

Developing countries such as Kenya still experience challenges around human resource to deliver refractive error services. However, given the burden of uncorrected refractive error, adoption of innovative and cost effective approaches is desirable. Hence this study intended to develop a task shifting framework integrated with telemedicine to potentially scale refractive error services.

**Methods:**

This was an exploratory study conducted in four phases as follows: a scoping review of the scope of practice for ophthalmic workers in Kenya, an interview with key opinion leaders on the need for integration of public health approaches such as the vision corridors within the eye health ecosystem in Kenya and their knowledge on task shifting, and finally development and validation of a proposed task shifting framework through a Delphi technique. Purposive sampling was used to recruit key opinion leaders and data was collected via telephonic interviews. The qualitative data was analyzed thematically using NVivo Software, Version 11.

**Results:**

The scoping review showed that only optometrists, ophthalmologists and ophthalmic clinical officers are allowed to undertake refraction in Kenya. All of the key opinion leaders (100%) were aware of task shifting and agreed that it is suitable for adoption within the eye health ecosystem in Kenya. All of the key opinion leaders (100%) agreed that skills development for healthcare workers without prior training on eye health supervised by optometrists through telemedicine is desirable. Notwithstanding, all of the key opinion leaders (100%) agreed that integration of public health approaches such as the vision corridors across all levels of healthcare delivery channels and development of a self-assessment visual acuity tool is desirable. Finally all of the key opinion leaders (100%) agreed that task shifting is relevant for adoption within the eye health ecosystem in Kenya. The developed framework prioritized partnership, advocacy, skills development, establishment and equipping of refraction points. The proposed framework advocated for a telemedicine between professionals with conventional training and those with skills development.

**Conclusion:**

Task shifting integrated with telemedicine could cost effectively scale refractive error service delivery. However, internal and external factors may hinder the success warranting the need for a multi-faceted interventions and a connection between planning and training to scale the uptake.

**Supplementary Information:**

The online version contains supplementary material available at 10.1186/s12913-024-10618-8.

## Background

Addressing uncorrected refractive error (URE) which is a type of vision problems that makes it hard to see clearly has been a major challenge in developing countries such as Kenya due to inadequate human resource [1]. Globally, it is estimated that approximately 2.2 billion people suffers from URE and it is projected to increase by 2050 [2]. It is projected that by 2050, half of the world population will suffer from myopia, 0.8 billion will suffer from hyperopia and 1.3 billion people will suffer from presbyopia [3]. However, given the evidence that only 10 − 20% of the population in need of refractive error (RE) services are able to access and afford the available RE services [4], action are desirable to ensure that the remaining proportion access and afford the available RE services. While in Kenya there are no population based studies on RE, approximately 6.39% are estimated to suffer from URE [5]. In Kenya, the Ministry of Health recommends one optometrist/ophthalmologist for a population of 250,000, one ophthalmic clinical officer for a population of 100,000 and one ophthalmic clinical officer/cataract surgeon for a population of 125,000 [6]. However, there are approximately 400 optometrists, 151 ophthalmologists, 40 ophthalmic clinical officers and 176 ophthalmic clinical officers/cataract surgeons whose current scope of practice allows them to undertake refraction to approximately 53 million Kenyans [6]. In consideration of the World Health Organization of one refractionist per 50,000 population [7], there is still a deficit of refractionists in Kenya warranting the need for adoption of cost effective approaches.

Awareness creation is an important component when it comes to scaling RE services. While developing countries such are Nigeria have adopted public health related approaches such as the vision corridors which is the self-assessment of the visual acuity by the general population in strategic places accessible to the URE [8], an evaluation of the impact of the approach is desirable to allow for replication in other settings. Currently in Kenya, the existing human resource has to stretch beyond their limits to deliver services while creating awareness concurrently. Considering that the scope of practice for eye care professionals destined to undertake refraction in Kenya does not include the aspect of awareness creation, adoption of holistic public health approaches are desirable. Therefore, this study evaluated the relevancy of the vision corridors approach within the eye health ecosystem in Kenya.

The World Health Organization (WHO) came up with a task shifting approach which is the redistribution of tasks among health workforce team members with an aim of scaling human resource in a cost effective way [9]. The concept is a leading and promising health system strategy to address health workforce shortage, transform healthcare delivery and improve health outcome and inequalities [10]. In countries such as Kenya, Malawi and Tanzania the concept has been adopted to address cataract which is the leading cause of unavoidable blindness globally [11]. According to Eliah et al., [12] task shifting for non-surgeons to address cataract surgery demands for quality training and allocation of more resources to ensure that cataract patients can be transported to a facility for surgery. Even though the study highlights that task shifting could potentially scale cataract services, there is an additional cost that is required to strengthen this approach. Another study by Gichangi et al., [13] on trichiasis also showed that task shifting may not effectively address the condition if quality of training is not strengthened. These studies are contrary to a study by Pente et al., [14] which reported that task shifting can address eye health under stringent policies. Even though adoption of task shifting maybe desirable for developing countries such as Kenya, the existing healthcare workforce remain limited and currently undertakes more tasks [11]. Conventionally, task shifting for paramedical teams such as the Community Health Volunteers (CHV) and Community Health Extension Workers (CHEW) in developing countries such as Kenya would be desirable; these cadres are already preoccupied with more tasks [15]. Therefore, considering that there is minimal information on task shifting and RE service delivery, this study intends to evaluate the potential of task shifting towards addressing URE.

In Kenya for instance, 90% of the eye care facilities offering RE services are located within urban areas with rural areas lacking refraction points which intern creates an imbalance in enhancing accessibility of RE across the economic pyramid [16, 17]. This shows a weak public health systems experiencing cost constraints in developing countries with majority of the eye care professionals undertaking refraction operating in urban areas [18]. However, with approximately 71% of Kenyans living in rural areas [17], integration of innovative approaches such as telemedicine which is the provision of health care remotely via information and communications technology is desirable [19]. According to the WHO, four out of five developing nations now offer at least one type of mobile health program to deliver essential health services to the population [18]. In Ethiopia, utilization of teleophthalmology has been recommended due to the huge ratio of 1 ophthalmologist to 1,200,000 patients [20]. Notwithstanding, mHealth has been shown as an approach with the potential of scaling services to remote areas, for instance the Peek Vision in Kenya has been shown to reduce avoidable blindness through early identification and improved adherence to referral [21]. While telemedicine is perceived to require an established infrastructure and technological resources to perform system wide functions, 89% of rural regions in South Africa, Mauritius, Kenya, and Malawi have access to the internet [22]. Telehealth applications could potentially increase operational and organizational efficiencies of existing systems, reduce health care costs and improve health outcomes even with limited resources [23]. While evidence shows the potential of telemedicine in the healthcare delivery [24], little information exists on the potential of the approach in supplementing other approaches such as task shifting. Although in Kenya, the concept of telemedicine was considered an alternative approach during Covid-19, the concept remains underutilized within the eye health ecosystem. Therefore, this study assessed the potential of telemedicine integration into the eye health ecosystem to strengthen task shifting.

## Methods

This study was conducted in four phases. The first phase entailed a scoping review of the scope of practice for ophthalmic workers in Kenya [6]. The scoping review was anchored on the WHO recommendations on the task shifting [9]. PubMed and Google Scholar were the main search engines used to extract information regarding scope of practice for ophthalmic workers in Kenya. Additional information was retrieved from the Ministry of Health website, National Strategic Plan for eye health 2020–2025 and policy documents for eye health. A Boolean operator of “AND” and “OR” was used. The keywords used were as follows: scope of practice OR refractive error OR ophthalmic workers OR refraction AND Kenya. The recommendations derived from the review were presented to key opinion leaders for an input.

In the second phase, the key opinion leader’s views were sought on the relevance of public health approaches such as the vision corridors in the eye health ecosystem in Kenya. With an assumption that awareness/education on RE among the general population remains low which significantly influences the uptake of the available RE services, getting the opinion of key leaders was deemed suitable [25, 26]. The key opinion leaders were purposively selected. The participants were recruited from eye care professionals associations, the private sectors, the Ministry of Information and Communication Technology, SE, the Ministry of Health and the public sectors. During recruitment of the participants, an email was sent to the organizations with details about the study requesting for a representative to participate.

A review of different levels of healthcare facilities in Kenya was undertaken with an intention to understand the current situation and to determine if task shifting could be justified in the Kenyan eye health ecosystem. PubMed and Google Scholar were the main search engines used to extract information regarding the levels of healthcare facilities in Kenya. Additional information was retrieved from the Ministry of Health website. A Boolean operator of “AND” and “OR” was used. The keywords used were as follows: primary level OR secondary level tertiary level OR healthcare AND Kenya.

The required cost to scale human resource through conventional training and through task shifting was undertaken. To determine the relevance of task shifting in addressing URE in Kenya, the cost required to bridge the human resource gap was estimated. The estimate was based on the current cost required to train an optometrist of between US$ 1,680 and US$ 7,000 [27, 28]. A holistic recommendation of task shifting through skills development was made. An assumption was made that even if cost effective RE services are integrated into the public health sectors, human resource remains critical for prioritization. At the same time, a suggestion was made that a strengthened public-private partnership is desirable.

During phase three, a task shifting framework was developed and validated through a Delphi technique with the key opinion leaders who had formed the sample for phase two. The development of the framework was based on the key opinion leaders’ inputs from phase one and two of the study. With the limited human resource in the general healthcare in Kenya [29], task shifting may not be holistic warranting skills development for competitively recruitment community members to undertake refraction. It was acknowledged that even though skills development is undertaken at a cost, it is cost effective when compared to conventional training. The first framework developed was based on the population density and primary care level facility. The second framework was modified to fit the current situation in Kenya and to scale awareness creation and vision screening for the public in a cost effective way. This could be achieved through competitive recruitment of community members for skills development.

Even though majority of the base of pyramid seek healthcare services from the public health sector due to affordability when compared to the private sectors, establishing refraction points within the public health sectors was proposed. The assumption was guided by the population density seeking healthcare services from the public health sectors facilities [30]. The task shifting framework subsequently developed was validated through key opinion leaders’ inputs using the Delphi technique.

During phase four, the developed task shifting framework was presented to 1,834 individuals comprising of community health volunteers (*n* = 1,023; 55.8%), clinical officers (*n* = 329; 17.9%) and nurses (*n* = 482; 26.3%) currently attached to different public and private health sectors in Kenya. The association representatives for the nurses, clinical officers and community health volunteers were contacted with details about the framework sent to them to disseminate to the association members. The key question accompanying the framework was whether the proposed framework is suitable for adoption in the Kenyan context and some inputs for its betterment (Supplementary material 1).

### Key opinion leader’s selection, recruitment and retention

Within our expert panel, it was determined that three Delphi rounds using email for correspondence would be sufficient to achieve consensus and stability [31, 32]. We aimed to retain a minimum of 10 key opinion leaders after three rounds of Delphi participation, and based on our experience with previous Delphi studies, we planned for 40% attrition in each round. To ensure that we achieve the minimum number of 10 key opinion leaders, we estimated that 35 invitees would be required in the first round of the Delphi. We adopted two approaches to convene the key opinion leaders engaged in eye care delivery in Kenya. The set of key opinion leaders recruited to the Delphi included a representative from the ophthalmic service unit Kenya, ophthalmologist representing the ophthalmological society of Kenya, optometrist representing the optometrists association of Kenya, Information and Communication Technology expert from an international SE, optometrist in-charge of training of the optical technicians, policy expert representative from the Kenya Society of the Blind, the head of partnership wider NGOs Africa from an international SE, ophthalmologists operating regional SE and a representative of the ophthalmic clinical officers. Rather than approaching the ophthalmic service unit Kenya alone, we chose a group of stakeholders in eye health working towards achieving universal health coverage to contribute to the development of the framework. Secondly, the key opinion leaders in our initial set of 35 contacts were invited to recommend colleagues who, in their opinion, might be interested in participating in this Delphi on the basis of their work or expertise. Throughout the Delphi process, key opinion leaders were blinded to the identity of others, except for individuals who referred us to subsequent key opinion leaders. Survey content was never associated with a key opinion leader identifier; only the researchers could associate key opinion leaders with responses. All the included questions from the survey concerned the respondents’ area of professional expertise. An email reminder was sent to key opinion leaders on a weekly basis to increase the response rate.

### Delphi rounds, data collection and analysis

A systematic and meaningful synthesis of responses was ensured through drafting and refining the questions asked the key opinion leaders in every round. The questionnaire was piloted among members of our authorship team who were not directly involved in designing the Delphi. We communicated with the key opinion leaders in English and used Google Forms to conduct our surveys.

A pre-specified definition of consensus was developed based on two criteria [33]. First, the key opinion leader was eligible to have achieved consensus around a given survey item if at least 70% of respondents agreed with that item. When using a five-point Likert scale, we defined “disagreement” as a score of two or less. This criterion ensured that a strong majority of respondents agreed with any included survey item. Second, an item was said to have achieved consensus only if none of the dissenting respondents raised concerns that were fundamentally incompatible with the inclusion of that survey item.

This criterion aligns with approaches from formal consensus decision-making, where a structured discussion is used to understand and resolve the merits and drawbacks of a given proposal [10]. This approach recognizes that essential insights can be tendered by a minority of decision-makers, and attends to the substance of minority opinions. Procedurally, these minority opinions were gathered by requiring that key opinion leaders offer free-text comments if they disapproved of a survey item. We analysed these free-text responses and incorporated that feedback into subsequent rounds of the Delphi and into the final task shifting framework. As the analysis advanced, an emphasis on and reiteration of certain issues above others became more apparent. These elements coalesced into the final categories and themes.

Round I was designed to elicit broad and general concepts from the key opinion leaders using unstructured, open-ended, questions:

1. What is, in your opinion, the purpose of task shifting?
2. What are the three to five characteristics of refractive error that make it amenable to task shifting?
3. What are three to five examples of delegation of responsibilities from highly qualified health workers to individuals with fewer qualifications and shorter training that are not task shifting?

Following Round I, the researchers combined and analysed the key opinion leader’s responses in taxonomy according to common themes and categories. We attempted to make the items on each list mutually exclusive and comprehensive. We synthesized these findings in a survey to elicit participants’ level of agreement with each of the themes and categories on a five-point Likert scale for Round II. This survey also offered free-text response options for key opinion leaders to add additional comments or categories as required.

Our study initially used the term “task shifting” in isolation, rather than the broader concepts of both task shifting and telemedicine. In Round I, key opinion leaders raised conceptual distinctions between task shifting and telemedicine. Recognizing that this had emerged organically from the Delphi process, we added questions to refine the distinction between task shifting and telemedicine and integrated this distinction into our conceptual model.

Once we received all responses from Round II, the data was again reviewed by the researchers. Concepts were eliminated and retained on the basis of the key opinion leader’s scores and collapsed into more general categories, including a definition of task shifting and telemedicine, the purpose of task shifting ad telemedicine, opportunities arising from task shifting and telemedicine programmes, and conditions required for the implementation of task shifting and telemedicine. These results were sent back to the key opinion leaders as a survey for Round III. Key opinion leaders were asked to review the final list of items, state whether they agreed or disagreed with each item, and voice concerns or comments in free text. Following Round III, the researchers integrated the experts’ consensus responses into a reasonable and manageable set of concepts and sub-concepts to form the framework [33].

The statistical data analysis for quantitative data was conducted in the Statistical Package for the Social Sciences version 29.0.0, 2022. The quantitative data was analysed through descriptive statistics of frequencies and percentages with data was presented using tables. For qualitative data, thematic analysis was carried out by categorizing the codes into categories using NVivo Software, Version 11 and themes based on the semantic meaning of the codes. It was an iterative process consisting of both deductive and inductive processes [34]. Initial codes and categories were generated from the interview guides (deductive process). New categories that consist of similar codes were added as required to capture the participants’ comments in details (inductive process). During this inductive process, the themes were identified by repetitions (the more the concept appears in the text, the more likely it is to be a theme), similarities and differences [35].

## Results

### Phase one

#### Review of the scope of practice for ophthalmic workers in Kenya

Currently, there are approximately 1,048 eye care professionals of different cadres in Kenya with 1,025 still required to meet the WHO recommendations on human resource to population ratio [6]. With sixteen cadres within the eye health ecosystem in Kenya who undertake various roles, only seven are allowed to undertake refraction with optometrists qualifying as clinical refractionists [6]. However, conventional training of optometrists may not be cost effective warranting the need for task shifting strengthened with telemedicine. Therefore, a task shifting approach should be undertaken for other cadres who are attached to primary and secondary level healthcare facilities supervised by optometrists through telemedicine. The other cadres that are well placed for task shifting are the optical technicians and the CHV. However, the current scope of practice for ophthalmic workers in Kenya limits them from undertaking refraction. Currently, the existing technicians and the CHV are preoccupied by other tasks, warranting the need for policies to advocate for skills development of community members to undertake refraction. This is anticipated to be cost effective when compared to conventional training and could potentially lessen the workload for the existing healthcare workforce.

All of the key opinion leaders (100%) agreed that current human resource in eye health in Kenya remains limited and the capacity cannot attend to the growing population in Kenya. The key opinion leaders argued that the suggestion of prioritizing skills development of competitively recruited community members to engage in refraction would potentially scale RE service in a cost effective way (quote 1).

1. *In my opinion, I will say this suggestion makes sense as training optometrists remains expensive and not only that, a lot of time is consumed. Hence if community members can be recruited competitively to engage in refraction then other cadres such as the community health volunteers and nurses will not be overburdened* - Opinion leader#04.

All of the key opinion leaders (100%) denoted that the current scope of practice for ophthalmic workers has not directed adequate attention towards addressing URE in Kenya. The key opinion leaders reported that the current cadres of ophthalmic workers in Kenya assign certain cadres more roles hence compromising the attention that the cadres direct towards addressing URE (quote 2).

2. *Although the magnitude of training determines the task one undertakes, I will agree with this proposed approach where a task shifting is applied for freshly trained individuals supervised by qualified eye care professionals* - Opinion leader#08.

### Phase two

#### Review of the of healthcare levels delivering refractive error services in Kenya

The government of Kenya is making efforts towards integrating eye health services within each and every level of healthcare delivery [36]. However, RE service delivery remains weak since the government depends majorly on human resource with conventional training as opposed to those with skills development warranting the need for task shifting. From the review, level 4 (County Hospitals), level 5 (County Referral Hospitals) and level 6 (National Referral Hospitals) healthcare facilities in Kenya comprises healthcare professionals who are allowed, as per the scope of practice, to undertake refraction. For level 1 (Community Facilities), level 2 (Health Dispensaries) and level 3 (Health Centres) healthcare facilities in Kenya, the existing cadres can only undertake basic ocular examination excluding RE service delivery. Hence, strengthening of level 1, 2 and 3 CHEWs through skills development inclined towards RE service delivery is desirable. A strengthened referral pathway should be established to ensure that CHEW with skills development can teleconsult optometrists for quality RE service delivery.

A third of the key opinion leaders (*n* = 3; 30%) agreed that undertaking skills development of competitively recruited community members could scale effective RE coverage (quote 3). However, all of the key opinion leaders (100%) agreed that integrating telemedicine would make quality RE service delivery (quote 4).

3. *Frankly refractive error can be scaled if the community members can be trained on the basics of dispensing and engage on community activities* - Opinion leader#03.
4. *I was a bit hesitant with the suggestion of skills development but now that they can be supervised through telemedicine then I agree it’s a brilliant suggestion that is worthy of attention*- Opinion leader#07.

From the review of the healthcare facilities in Kenya at each level where RE service could be integrated, there are cumulatively 12,393 facilities of different levels existing in Kenya with the public sector consituting the highest followed by the private sectors [36]. This justifies that the private sector play a crucial role in the healthcare delivery in Kenya. Integration of public health approaches such as the vision corridors in each and every healthcare level in Kenya is desirable to scale awareness.

Two third of the key opinion leaders (*n* = 6; 60%) agreed that integration of RE services across all sectors would scale RE service delivery. However a third of the key opinion leaders (*n* = 3; 30%) denoted that integrating RE service across all levels of healthcare delivery may not be realistic given the difference in the population distribution where a healthcare facility is located (quote 5).

5. *Given the variation in the population distribution within the geographical location of a health facility, I tend to think the best thing we can do is to integrate RE services in like level 3 and above but we also make some levels to focus more on awareness creation and community activities*- Opinion leader#03.

In Kenya, there are 11,547 healthcare facilities at level 2 and 3, 839 healthcare facilities at level 4 and 5 and finally 7 healthcare facilities at level 6 [36]. Therefore, if each level 2 and 3 healthcare facility is to be managed by an optometrist then approximately 11,147 will be required given that approximately 400 optometrists currently exists in Kenya [36]. Hence level 4 and 5 healthcare facilities should be targeted given the larger catchment area. Training an optometrist in Kenya cost approximately US$ 7,000 while skills development cost approximately US$ 1000 [37]. Therefore, prioritizing skills development as a form of task shifting would be cost effective.

All of the key opinion leaders agreed that training optometrists would be more ideal, however, skills development as a form of task shifting for competitively recruited community members would be cost effective (quote 6).

6. *I agree that training optometrists would be the best option, but given the limited resources available, the suggestion of skills development as a form of task shifting I think is more relevant but training of optometrists should also be done concurrently*- Opinion leader#07.

### The vision corridors

Given the challenges around accessibility and availability of RE services within the eye health ecosystem in developing countries such as Kenya [38], integration of vision corridors was proposed.

Almost three quarters of the key opinion leaders (*n* = 9; 90%) were aware of the vision corridors concept and agreed that the concept is relevant for the eye health ecosystem in Kenya. The challenge reported by the key opinion leaders was how the public would interpret their visual acuity scores (quote 7). The key opinion leaders argued that task shifting for community members with skills development to supervise the vision corridors would make the approach more suitable and increase the likelihood of referral for refraction (quote 8).

7. *I have always thought this vision corridors approach is necessary in Kenya and if it can be integrated in the eye health ecosystem then supervision is desirable to help the public interpret*- Opinion leader#05.
8. *Vision corridors approach is good but I think it can be much better if there is an individual with basic training guiding the individuals and referring them for refraction-* Opinion leader#07.

### Phase three

#### The key opinion leader’s perspective on task shifting

All of the key opinion leaders (100%) were aware of the task shifting concept and agreed that it is suitable for adoption within the eye health ecosystem in Kenya. With existing policies around the healthcare workforce in Kenya, the key opinion leaders (100%) agreed that establishment of policies is desirable to recognize skills development as a form of task shifting within the eye health ecosystem.

### A possible task shifting framework for addressing uncorrected refractive error in Kenya

**Fig. 1:**
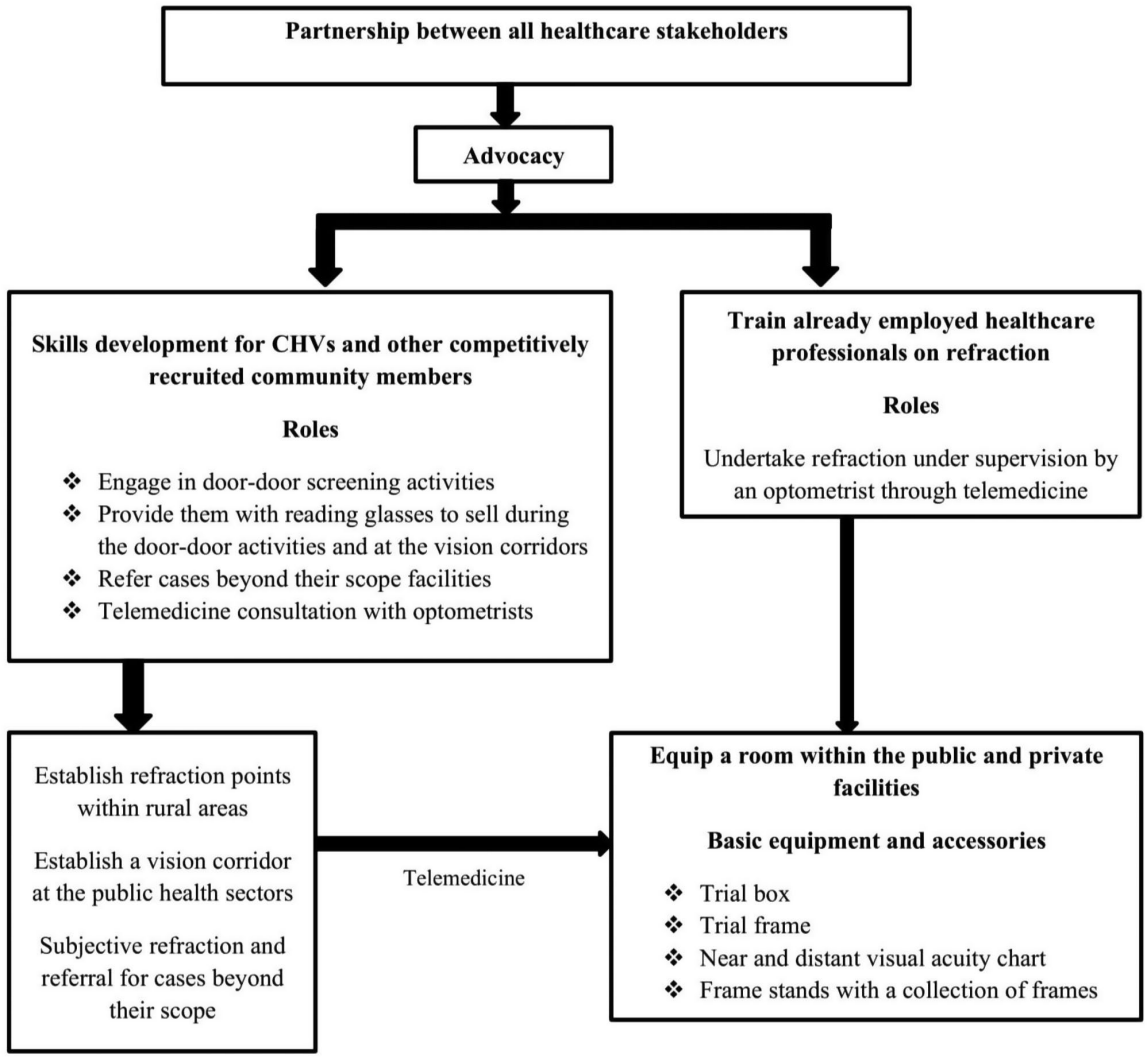
A possible task shifting framework for addressing uncorrected refractive error in Kenya

This proposed task shifting framework is developed for primary level facilities with low population having healthcare workers and CHV with minimal tasks. The proposed framework will be utilized across the private and public sectors. The key aspects prioritized in the development of this proposed framework included partnership, advocacy, policies, skills development and referral. The proposed framework is shown in Fig. 1 and a brief description of the content thereafter.

This proposed task shifting framework will entail a partnership between the stakeholders in eye health and the targeted healthcare cadre working in primary level healthcare facilities in geographical areas with low population. The core reason for targeting primary care level facilities is to ensure that RE are made available in rural areas as such facilities are located in such geographical locations. Skills development should be integrated into existing training institutions offering eye health courses.

All of the key opinion leaders (100%) agreed that the suggestion to advocate for task shifting within primary care facilities is desirable to scale RE services to such underserved population (quote 9).

9. *We have had problems with human resource in primary care facilities and a suggestion to undertake task shifting is justifiable-* Opinion leader#06.

This framework also advocates for mobilization of resources intended to equip healthcare facilities at the primary care level facilities. All of the key opinion leaders (100%) agreed that the suggestion of equipping the primary care level health facilities will scale availability and accessibility (quote 10).

10. *The main challenge we have is the aspect around accessibility and availability of refractive error services. Hence the recommendation for advocating for eye care services within primary facilities is ideal* - Opinion leader#03.

The optical technicians should engage in rural vision screening in the established satellite refraction points under supervision by an optometrist through telemedicine. The refraction points established in the rural areas should integrate the vision corridor approach.

All of the key opinion leaders (100%) agreed that establishing refraction points in rural areas is desirable and relevant provided a channel is designed to supervise their activities (quote 11).

11. *In my view the suggestion of allowing optical technicians to establish refraction points in rural areas is worth as many are unable to access refractive error services from such regions. But they should be supervised to ensure they do the right thing*- Opinion leader#01.

Since majority of patients who visit health care facilities are not aware of their RE status, integration of the vision corridors approach across public and private health facilities was proposed. This is intended to create awareness among patients visiting healthcare facilities on their RE status.

All of the key opinion leaders (100%) denoted that the suggestion to incorporate vision corridors across all public and private healthcare facilities is ideal in enhancing awareness in a cost effective way (quote 12).

12. *I think hospitals receive volumes of people such that recording visual acuity for everyone may not be realistic and I endorse the vision corridor approach suggestion as it will scale awareness for everyone who visit an hospital*- Opinion leader#07.

Considering that limited resources is a major challenge in the healthcare sector in Kenya, all of the key opinion leaders (100%) argued that this proposed task shifting framework is good but would benefit from modification to suit the current situation in a more holistic way (quote 13).

13. *This proposed framework is good but requires resources which are not readily available; as a result I would say it should be modified to be cost effective*- Opinion leader#08.

### Modification of a proposed holistic task shifting framework for scaling human resource to address uncorrected refractive error in Kenya

**Fig. 2:**
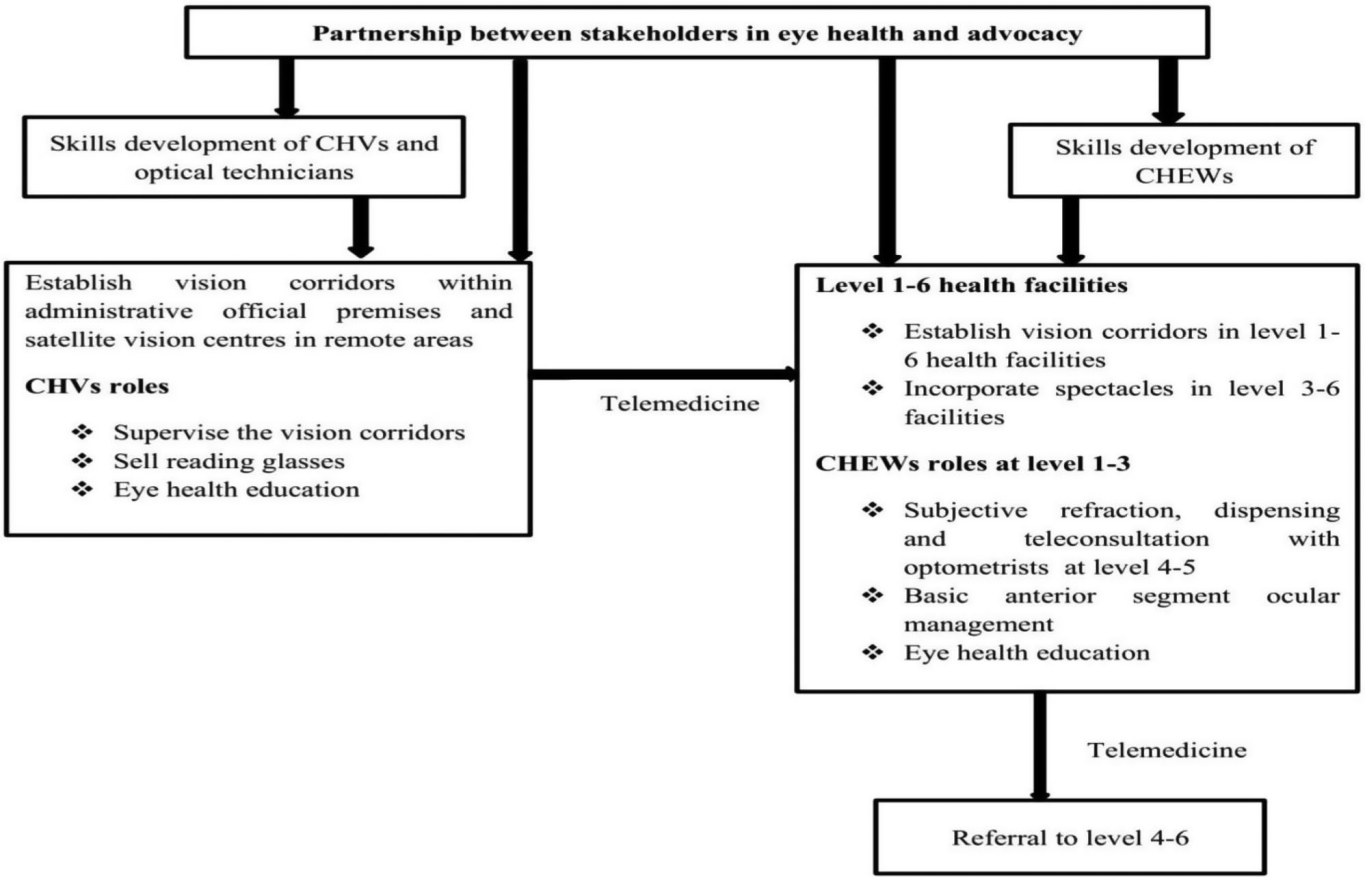
Modified proposed holistic task shifting framework for scaling human resource to address uncorrected refractive error in Kenya

Based on the key opinion leaders’ inputs, the task shifting framework was modified as shown in Fig. 2 and expounded thereafter.

The key aspect to be considered for the success of this proposed framework is partnership and advocacy among stakeholders in eye health and other cadres. The partnership and advocacy is projected to scale awareness and enhances a team approach among the existing eye care professionals and the healthcare providers with extended duties through task shifting. Self-assessment for visual acuity was prioritized as a key important aspect that should be operationalized. Aspects such as telereferral and teleconsultation should be strengthened.

All of the key opinion leaders (100%) agreed that the content of this proposed task shifting framework is adequate for a developing country such as Kenya. The key opinion leaders argued that the proposed framework has the potential to scale RE service delivery (quote 14).

14. *I think for a start, this framework is good for Kenya as it addresses the challenges around accessibility, availability and affordability in a cost effective way, hence I think if adopted can create a difference in the eye health ecosystem*- Opinion leader#07.

### Phase four

#### Nurses, clinical officers and community health volunteer’s perspective on the proposed framework

All of the nurses, clinical officers and community health volunteers (100%) reported that what makes the proposed framework holistic is the aspect of telemedicine integration (quotes 15–16).

15. *The main concern that makes task shifting unsuccessful is due to the fact that healthcare workers with skills development have to also engage in their activities hence the integration of telemedicine will ensure that the quality of service delivery is strengthened-* Clinical officer.
16. *Normally, task shifting remains unrecognized in many health facilities in Kenya hence through telemedicine, there will be recognition that the roles undertaken by the general healthcare workers remain recognized and are properly supervised***-** Nurse.

All of the nurses, clinical officers and community health volunteers (100%) acknowledged that even though the proposed task shifting approach is desirable, they reported that challenges will be experienced during implementation as they are currently limited in number and are overburdened by their core activities (quote 17).

17. *As it stands, we are currently limited in number with more tasks that additional tasks may hinder us from undertaking our roles effectively*- Nurse.

## Discussion

The chronic shortage and uneven distribution of eye care professionals in Sub-Saharan Africa has resulted to a number of countries training primary healthcare professionals to undertake various roles [32]. This is called task shifting which has greatly been advocated for by the WHO to aid in addressing various health conditions in countries with limited workforce. The WHO has devised global recommendation and guideline on the concept to support and guide the expansion of healthcare initiatives and workforce organizations in countries [39]. This framework intends to establish conditions for safe, equitable, efficient, effective and sustainable task shifting. Various studies have shown that task shifting demands that healthcare workers should receive training and continuous medical education for effective delivery [10, 40]. This study has shown that task shifting for competitively recruited community members through skills development could enhance efficiency and reduce the cost associated with RE services. The results are similar to a study by Antwi-Boasiako & Machin, [41] which reported that task shifting through utilization of primary healthcare enhances efficiency in service delivery. Even though task shifting has proven to be cost effective in scaling human resource to attend to the growing population of HIV-infected patients, minimal information exists on the approach in addressing URE [32]. Therefore, this study has shown that this approach would be ideal for developing countries such as Kenya with limited human resource that are unevenly distributed.

In Eastern Africa, the concept of task shifting has been adopted to address trichiasis and cataract [13]. A study by Gichangi et al., [13] reported that task shifting for trichiasis surgery is not reasonable approach to eliminate blinding trachoma as training of the middle level healthcare professionals as trachomatous trichiasis surgeons does not include provision of surgical set to address the backlog. Another study by Eliah et al., [11] reported that task shifting requires high quality training but is not sufficient to result in cataract surgical activities that meet population needs. This study has shown that individuals with skills development should constantly be supervised through telemedicine so as to strengthen the quality and RE patient satisfaction. In a review of 34 studies on the possible cost saving of task shifting, 30 studies revealed a drop in health cost due to adoption of task shifting to health system and patients [42]. Another study by Seidman & Atun, [43] reported that task shifting improves quality of patient care through reduced wait time, increased access to care and reduced patient mortality. Therefore, to address URE through task shifting, aspects around value in training, support and supervision for the dedicated teams should be addressed through continuity between planning and training on. In Nigeria, a study by Adejumo et al., [44] reported that more than half of the physicians had a good perception of task shifting with about two-third believed that it could be implemented successfully. While this study did not assess the productivity and RE quality outcome through task shifting, such studies are desirable.

Currently in Kenya, there are no policies outlining how task shifting should be undertaken to address URE [40]. While the National Strategic Plan for eye health 2020–2025 has documented task shifting, minimal information on how it should be undertaken. However, evidence has shown that effective implementation of task shifting will require funding, planning, training and education [45, 46]. Hence this study has provided a possible framework that could be adopted through cross-sectorial collaboration. The findings are similar to a study by Gichangi et al., [13] which reported task shifting towards addressing trachoma demands for collaboration across all sectors engaged in eye care delivery. This study has also showed that majority of the stakeholders in eye health in Kenya are familiar with the concept of task shifting and agree that the concept is suitable for adoption within the eye health ecosystem in Kenya. A study by Yankam et al. [32], reported that characteristics of staff that is accessible, the health issue and the intervention are some of the conditions that must exists for a successful task shifting. Again in Low Middle Income Countries, a study by Le et al. [47], showed that patient characteristics such as the level of education are a key barrier to task shifting intervention. This study acknowledges that task shifting may experience challenges such attrition by the primary healthcare professionals due to overburden and the cost required for skills development. However, it proved cost effective compared to conventional training. Therefore, the policy environment should be re-engineered to align with the proposed framework and advocacy efforts should be strengthened to ensure training of the relevant cadres as per the scope of practice, and building the appropriate health system. Notwithstanding, future studies are desirable to generate evidence on the cost effectiveness of task shifting approach in addressing URE.

According to the World Health Organization task shifting may only be effective if other efforts are integrated with the approach [9]. While some studies on task shifting have shown that the concept has been adopted solely to address trachoma and cataract [13], the supervision is up-taken by ophthalmologists through face to face approach. This study has shown that integration of telemedicine would be suitable in supporting task shifting. A meeting abstract by Mair & Whitten, [49] examining telemedicine delivered subjective refraction found no statistically significant difference between in-person and telemedicine delivered subjective refraction. Evidence shows that in primary care, telemedicine facilitates communication with the general practitioners, improve access and treatment for elderly thus reducing the healthcare cost [48]. Notwithstanding, with variation in the socioeconomic status, telemedicine has provided healthcare to previously underserved regions with provision of care which was not previously deliverable [48]. Therefore, this study proposes for utilization of telemedicine by the primary healthcare individuals with task shifting operating in the underserved remote areas.

Telemedicine has been applied within remote settings to overcome the geographical barriers to healthcare access and provision of alternative means of connecting patients to specialists [50]. This current study proposes utilization of telemedicine to scale RE services to remote underserved areas through consultation between eye care professionals and individuals with skills development. The use of telemedicine has been supported and regarded to be cost effective, reliable and valid in eye health delivery [51, 52]. A study on cost effectiveness of optometry facilitated telehealth services demonstrated the delivery of low vision telerehabilitation significantly reduced the cost to the patient and increased access to low vision services compared to in person services [53]. Given that minimal information exists on the cost of establishing telemedicine that could be utilized directly between patients and the eye care professionals, this study advocates for a consultation between eye care professionals with conventional training and those with skills development. Therefore, adopting an integrated approach for task shifting and telemedicine could potentially address URE. While evidence shows that most governments have not implemented the concept of telemedicine, patients are satisfied with quality of care [53]. Therefore, with the enormous benefits from telemedicine, advocacy towards recognition of the concept and assessment of the patient view on the concept are desirable. Notwithstanding, a cost benefit analysis of the concept is desirable in future studies.

Although telemedicine in eye health is a well-researched discipline and highly regarded by practitioners [53], this study has identified financial, technical and logistical obstacles as key barriers with the approach. This study results are similar to a study by Kim & Zuckerman, [22] on realizing the potential of telemedicine in global health. Legally, the concept of telemedicine has been shown to be associated by drawbacks such as liability, responsibility and accountability [53]. Notwithstanding, the risk of a third party intercepting communication by accessing an electronic medical record is a drawback of telemedicine [53]. Hence this study recommends for establishment of policies to regulate the concept just like in the Unites States and United Kingdom where doctors practicing telemedicine are bound by similar duty of care just like face to face [54]. Therefore, addressing these challenges linked with telemedicine demands for application of multifaceted approach to scale awareness, educational resources, logistical support and funding.

### Electronic supplementary material

Below is the link to the electronic supplementary material.

Supplementary Material 1

## Data Availability

The data is available upon reasonable request from the corresponding author (Email: mumashadrack275@gmail.com).

## References

[1] Patel D, Gilbert S (2018). Investment in human resources improves eye health for all. Community Eye Heal J.

[2] Bourne RRA, Steinmetz JD, Saylan M, Mersha AM, Weldemariam AH, Wondmeneh TG (2021). Causes of blindness and vision impairment in 2020 and trends over 30 years, and prevalence of avoidable blindness in relation to VISION 2020: the right to Sight: an analysis for the global burden of Disease Study. Lancet Glob Heal.

[3] Holden BA, Fricke TR, Wilson DA, Jong M, Naidoo KS, Sankaridurg P (2016). Global prevalence of myopia and high myopia and temporal trends from 2000 through 2050. Ophthalmology.

[4] Hashemi H, Fotouhi A, Yekta A, Pakzad R, Ostadimoghaddam H. Global and regional estimates of prevalence of refractive errors: Systematic review and meta-analysis. J Curr Ophthalmol [Internet]. 2018;30(1):3–22. 10.1016/j.joco.2017.08.009. 10.1016/j.joco.2017.08.009 PMC5859285 29564404

[5] Muma S, Naidoo KS, Hansraj R. Estimation of the prevalence of refractive error in Kenya: a systematic review and Meta-analysis. Optom Vis Perform. 2023;11(3).

[6] Kenya Ministry of Health. Scope of practice for ophthalmic workers [Internet]. 2022. Available from: https://www.health.go.ke/.

[7] Raman U. Human resources for eye care: changing the way we think. Community Eye Heal [Internet]. 2009 Mar [cited 2021 Oct 4];22(69):12. Available from: https://www.pmc/articles/PMC2683557. PMC2683557 19506715

[8] Ugalahi MO, Bekibele CO, Ogundipe AO. Utilization of Vision Corridor for Self-evaluation of Vision Among Secondary School Students in Igbo-Ora, Southwest. 2023;39–44.

[9] World Health Organization. Task Shifting: Global Recommendations and Guidelines. 2008.

[10] Orkin AM, Rao S, Venugopal J, Kithulegoda N, Wegier P, Ritchie SD et al. Conceptual framework for task shifting and task sharing: an international Delphi study. Hum Resour Health [Internet]. 2021;19(1):1–8. 10.1186/s12960-021-00605-z. 10.1186/s12960-021-00605-z PMC8091141 33941191

[11] Eliah E, Lewallen S, Kalua K, Courtright P, Gichangi M, Bassett K (2014). Task shifting for cataract surgery in eastern Africa: Productivity and attrition of non-physician cataract surgeons in Kenya, Malawi and Tanzania. Hum Resour Health.

[12] Eliah E, Lewallen S, Kalua K, Courtright P, Gichangi M, Bassett K. Task shifting for cataract surgery in eastern Africa: Productivity and attrition of non-physician cataract surgeons in Kenya, Malawi and Tanzania. Hum Resour Health [Internet]. 2014;12(1):S4. Available from: http://www.human-resources-health.com/content/12/S1/S4. 10.1186/1478-4491-12-S1-S4 PMC4108920 25859627

[13] Gichangi M, Kalua K, Barassa E, Eliah E, Lewallen S, Courtright P (2015). Task shifting for Eye Care in Eastern Africa: General nurses as Trichiasis surgeons in Kenya, Malawi, and Tanzania. Ophthalmic Epidemiol.

[14] Pente V, Tobi P, Roca A, Ogun- K. Task-shifting eye care to ophthalmic community health officers (OCHO) in Sierra Leone: A qualitative study. 2021;(March). 10.7189/jogh.11.07001 PMC7956140 33763216

[15] Mulaki A, Muchiri S. Kenya Health System Assessment. 2019.

[16] Palmer JJ, Chinanayi F, Gilbert A, Pillay D, Fox S, Jaggernath J et al. Mapping human resources for eye health in 21 countries of sub-saharan Africa: current progress towards VISION 2020. Hum Resour Health. 2014;12(1). 10.1186/1478-4491-12-44 PMC4237800 25128163

[17] Courtright P, Mathenge W, Kello AB, Cook C, Kalua K, Lewallen S. Setting targets for human resources for eye health in sub-saharan Africa: what evidence should be used? Volume 14. Human Resources for Health. BioMed Central Ltd.; 2016. 10.1186/s12960-016-0107-x PMC4794905 26984773

[18] Du Toit R, Faal HB, Etya’Ale D, Wiafe B, Mason I, Graham R et al. Evidence for integrating eye health into primary health care in Africa: a health systems strengthening approach. BMC Health Serv Res. 2013;13(1). 10.1186/1472-6963-13-102 PMC3616885 23506686

[19] Sood S, Mbarika V, Jugoo S, Dookhy R, Doarn CR, Prakash N (2007). What is telemedicine? A collection of 104 peer-reviewed perspectives and theoretical underpinnings. Telemed e-Health.

[20] Mbarika VWA, Datta P, Media S. Telemedicine in Sub-Saharan Africa: The Case of Teleophthalmology and Eye Care in Ethiopia. 2006;(November 2019).

[21] Rono H, Bastawrous A, MacLeod D, Wanjala E, Gichuhi S, Burton M. Peek Community Eye Health - MHealth system to increase access and efficiency of eye health services in Trans Nzoia County, Kenya: study protocol for a cluster randomised controlled trial. Trials. 2019;20(1). 10.1186/s13063-019-3615-x PMC6694474 31412937

[22] Kim T, Zuckerman JE (2019). Realizing the potential of telemedicine in global health. J Glob Health.

[23] Kruse CS, Krowski N, Rodriguez B, Tran L, Vela J, Brooks M (2017). Telehealth and patient satisfaction: a systematic review and narrative analysis. BMJ Open.

[24] Dzando G, Akpeke H, Kumah A, Agada E, Lartey A, Nortu J et al. Telemedicine in Ghana: insight into the past and present, a narrative review of literature amidst the Coronavirus pandemic. J Public Health Africa. 2022;13(1). 10.4081/jphia.2022.2024 PMC9202481 35720800

[25] Fred Hollows Foundation. The Power of Impact Investment to Improve Vision. 2015.

[26] Johansen AS, Vracko P, West R. The evolution of community-based primary health care, Slovenia. Bulletin of the World Health Organization. Volume 98. World Health Organization; 2020. pp. 353–9. 10.2471/BLT.19.239616 PMC7265942 32514200

[27] Kenya Medical Training College. Kenya Medical Training College | Training For Better Health [Internet]. 2022. Available from: https://kmtc.ac.ke/.

[28] Masinde Muliro University. Bachelor of Optometry fee structure [Internet]. 2019. p. 597360. Available from: https://www.mmust.ac.ke/index.php/student-fee-structures.

[29] Kimathi L (2017). Challenges of the Devolved Health Sector in Kenya: teething problems or systemic contradictions?.

[30] KNBS. Distribution of Population by Administrative Units [Internet]. Vol. II, 2019 Kenya Population and Housing Census. 2019. 251 p. Available from: http://www.knbs.or.ke.

[31] Burn H, Black J, Harwood M, Gordon I, Burnett AM, Hamm LM, et al. Eye care delivery models to improve access to eye care for indigenous people in high-income countries: protocol for a scoping review. BMJ Open. Volume 9. BMJ Publishing Group; 2019. 10.1136/bmjopen-2019-029214 PMC6677968 31362967

[32] Yankam BM, Adeagbo O, Amu H, Dowou RK, Nyamen BGM, Ubechu SC et al. Task shifting and task sharing in the health sector in sub-saharan Africa: evidence, success indicators, challenges, and opportunities. Pan Afr Med J. 2023;46. 10.11604/pamj.2023.46.11.40984 PMC10683172 38035152

[33] Niederberger M. Delphi Technique in Health Sciences: Front Public Heal. 2020;8(September):1–10. 10.3389/fpubh.2020.00457 PMC7536299 33072683

[34] Fereday J, Muir-Cochrane E (2014). Demonstrating Rigor using thematic analysis: a Hybrid Approach of Inductive and deductive coding and theme development. Conv Prev Punishm Crime Genocide.

[35] Ryan GW, Bernard HR. Techniques to Identify Themes. Field methods [Internet]. 2003;15(1):85–109. 10.1177/1525822X02239569.

[36] Kenya Ministry of Health. National Eye Health Strategic Plan [Internet]. 2020. Available from: https://www.medbox.org/document/national-eye-health-strategic-plan-2020-2025.

[37] Essilor, EYE RAFIKI - Essilor See Change [Internet]. 2018 [cited 2023 Aug 19]. Available from: https://www.essilorseechange.com/what-we-do/2-5-new-vision-generation/eye-rafiki/.

[38] Sengo DB, Marraca NA, Muaprato AM, García-Sanjuan S, Caballero P, López-Izquierdo I. Barriers to Accessing Eye Health Services in Suburban communities in Nampula, Mozambique. Int J Environ Res Public Health. 2022;19(7). 10.3390/ijerph19073916 PMC8997994 35409600

[39] Okyere E, Mwanri L, Ward P. Is task-shifting a solution to the health workers’ shortage in Northern Ghana? PLoS One [Internet]. 2017;12(3):e0174631. 10.1371/journal.pone.0174631. 10.1371/journal.pone.0174631 PMC5373592 28358841

[40] Kinuthia R, Verani A, Gross J, Kiriinya R, Hepburn K, Kioko J et al. The development of task sharing policy and guidelines in Kenya. Hum Resour Health [Internet]. 2022;20(1):1–12. 10.1186/s12960-022-00751-y. 10.1186/s12960-022-00751-y PMC9336004 35906629

[41] Antwi-Boasiako S, Machin H (2020). Ophthalmic nurses: vital team members in the push for better eye health. Community eye Heal.

[42] Mdege ND, Chindove S, Ali S. The effectiveness and cost implications of task-shifting in the delivery of antiretroviral therapy to HIV-infected patients: a systematic review. 2012;(June 2018). 10.1093/heapol/czs058 22738755

[43] Seidman G, Atun R (2017). Does task shifting yield cost savings and improve efficiency for health systems? A systematic review of evidence from low-income and middle-income countries. Hum Resour Health.

[44] Adejumo OA, Ogundele OA, Mamven M, Otubogun FM, Junaid OA, Okoye OC et al. Physicians’ perception of task sharing with non-physician health care workers in the management of uncomplicated hypertension in Nigeria: A mixed method study. PLoS One [Internet]. 2023;18(9 September):1–16. 10.1371/journal.pone.0291541. 10.1371/journal.pone.0291541 PMC10529560 37756324

[45] van Schalkwyk MC, Bourek A, Kringos DS, Siciliani L, Barry MM, De Maeseneer J (2020). The best person (or machine) for the job: rethinking task shifting in healthcare. Health Policy.

[46] Colvin CJ, de Heer J, Winterton L, Mellenkamp M, Glenton C, Noyes J (2013). A systematic review of qualitative evidence on barriers and facilitators to the implementation of task-shifting in midwifery services. Midwifery.

[47] Le PD, Eschliman EL, Grivel MM, Tang J, Cho YG, Yang X et al. Barriers and facilitators to implementation of evidence-based task-sharing mental health interventions in low- and middle-income countries: a systematic review using implementation science frameworks. Implement Sci [Internet]. 2022;17(1):4. 10.1186/s13012-021-01179-z. 10.1186/s13012-021-01179-z PMC8756725 35022081

[48] Mair F, Whitten P (2000). Systematic review of studies of patient satisfaction with telemedicine. BMJ.

[49] Mair F, Whitten P (2000). Systematic review of studies of patient satisfaction with telemedicine. Br Med J.

[50] Chia MA, Turner AW (2022). Benefits of integrating Telemedicine and Artificial Intelligence Into Outreach Eye Care: Stepwise Approach and future directions. Front Med.

[51] Labiris G, Panagiotopoulou EK, Kozobolis VP (2018). A systematic review of teleophthalmological studies in Europe. Int J Ophthalmol.

[52] Tan IJ, Dobson LP, Bartnik S, Muir J, Turner AW (2017). Real-time teleophthalmology versus face-to-face consultation: a systematic review. J Telemed Telecare.

[53] Ihrig C (2019). Travel cost savings and practicality for low-vision telerehabilitation. Telemed J e-health off J Am Telemed Assoc.

[54] Kim T, Zuckerman JE. Realizing the potential of telemedicine in global health. J Glob Health. 2019;9(2). 10.7189/jogh.09.020307 PMC6790231 31656598

