# Supplementary material for: Proposed task shifting integrated with telemedicine to address uncorrected refractive error in Kenya: Delphi study
Source: BMC Health Serv Res. 2024 Jan 22;24:115. doi: 10.1186/s12913-024-10618-8 (PMC10801974; doi:10.1186/s12913-024-10618-8)
Supplement: Supplementary file 1 — Supplementary Material 1 [file 12913_2024_10618_MOESM1_ESM.docx]

PHASE ONE

1. What is the current human resource in the eye health ecosystem in Kenya?
2. What is the refractive error need in Kenya based on the population and the existing human resource?
3. What is the potential of the scope of practice for ophthalmic workers in Kenya to meet the RE needs?
4. What are the gaps within the scope of practice for ophthalmic workers in Kenya?
5. To what extent does task shifting exists within the eye health ecosystem in Kenya?
6. Can task shifting be undertaken within the existing cadres in the eye health ecosystem?
7. Does quality assurance exist within the eye health ecosystem in Kenya?

PHASE TWO

1. What is the relevance of public health approaches such as the vision corridors in the eye health ecosystem in Kenya.
2. What is the relevance of telemedicine integration into the eye health ecosystem in Kenya?
3. What is the scope of practice for the available human resource in Kenya suitable for skills development to undertake refraction?
4. Are refractive error services available within the public health sectors in Kenya?
5. How many public health facilities in Kenya lack refractive error services?
6. What is the cost required to bridge the human resource gap if a clinical refractionists should be present in each and every identified public health facility without optometrists?

PHASE THREE

1. What is your view on the proposed task shifting framework?

PHASE FOUR

1. Is the proposed framework suitable for adoption in the Kenyan context?
2. What are some of the inputs that you think may strengthen the proposed framework?
